# Supplementary material for: Evaluation of methods for the measurement of antibody-dependent enhancement of dengue virus infection using different FcγRIIa expressing cell lines
Source: PLoS One. 2025 Aug 29;20(8):e0331320. doi: 10.1371/journal.pone.0331320 (PMC12396659; doi:10.1371/journal.pone.0331320)
Supplement: Attachment — Submitted filename: Response to reviewers.docx [file pone.0331320.s001.docx]

Dear Editor and Reviewers,

Thank you for your valuable feedback on our manuscript titled "Comparative evaluation of methods for the measurement of antibody-dependent enhancement of dengue virus infection in FcγRIIa expressing cell lines" (Manuscript ID: PONE-D-24-39295 PLOS ONE). We appreciate the time and effort you have invested in reviewing our work. We have carefully considered all the comments and have made significant revisions to address them.

**Response to the Academic editor:**

1. Please carefully revise your manuscript according to the reviewers' comments and suggestions.

**Response:** We have addressed each comment and revised the manuscript accordingly.

2. Please double-check your manuscript, and address the following issues:

(1) To find expert or company to polish the English of your manuscript.

**Response:** We have tried to improvise our writing language for better clarity.

(2) To avoid missing text (for example, in line 43, "cell" missing after "Vero" and "Vero-CD32a").

**Response:** We have added missing text throughout the manuscript.

(3) The results section of the abstract did not indicate that K562 cells were superior to U937 cells in evaluating ADE.

**Response:** We have revised the results section of the abstract highlighting the superiority of K562 cells over U937 cells in evaluating ADE.

(4) In fact, a large number of studies have compared and evaluated the advantages and disadvantages of various cell lines in evaluating ADE. Please make appropriate citations in the article and compare them with your manuscript.

**Response:** We have compared our results with published literature by evaluating the different FcγR expressing cell lines in the revised manuscript (Line no. 566 – 582, page no. 26).

(5) The title of the article does not match the content of the evaluation of ADE and neutralization experiments on different cell lines.

**Response:** We've made changes to the title.

(6) Plaque Assay (PA) should be replaced with Focus-forming assay (FFA). Virus titer should be expressed as focus-forming unit (FFU) per ml rather than PFU. Why is a culture medium containing aquacide used to cover cells in this experiment? Generally, a culture medium containing methylcellulose or low melting point agar is used to cover cells.

**Response:** Several papers in the literature make use of both plaque assay (PA) or focus-forming assay (FFA) and plaque-forming unit (PFU) per ml or focus-forming unit (FFU) per ml as inter-changeable terminologies. We have made the changes as suggested by the reviewer. Also, we have indicated aquacide as the overlay medium used in the manuscript; as aquacide is a commercial name for carboxymethylcellulose. We have reworded the sentence in the revised manuscript (Line no. 173, page no. 8).

(7) The pan-flavivirus 4G2 antibody was used in the ADE assay, but the antibody HB112 appears in the experimental results section and figures.

**Response:** Pan-flavivirus 4G2 antibody is also referred to as HB112 antibody which is the ID of the clone from which it is derived. We have indicated pan-flavivirus 4G2 antibody as HB112 antibody throughout the text.

**Response to the Reviewer 1:**

Reviewer #1: This manuscript written by Chelluboina et al. describes a basic study on the evaluating ADE activity against dengue virus. Authors compared some protocols of ADE assay using several Fcγ receptor-expressing cell lines (K562, U937 and Vero-CD32a cells). They also compared neutralizing activities between normal Vero and Vero-CD32a cells. They concluded that K562 cells were the most suitable to be used for evaluating ADE activity against dengue virus, and Vero-CD32a cells showed significantly lower neutralizing activity than normal Vero cells when DENV-2 was used as an assay antigen. Possibly the experimental design was elementary, thus the data were not exciting like I have ever seen before. I could not find any unique points which differentiated from other studies. Specific comments are below.

**Response:** Thank you for the comments. Although several studies have been done using FcγR-expressing cell lines, here we present an evaluation of two commonly used methods to measure ADE response and compare our results with published literature by evaluating the different FcγR expressing cell lines (Line no. 566 – 582, page no. 26).

We have added a new Table 3, that clearly demonstrates the sub-neutralizing levels of antibodies leading to antibody enhancement and peak enhancement at different dilutions varied among four serotypes of DENV.

Major comments

(1) Lines 132-135: Information of the Vero-CD32a cells is needed further. Please add a reference describing the origin of Vero-CD32a cells.

**Response:** Vero cells expressing CD32a receptor (Vero-CD32a cells) were received from Dr. Stephen Whitehead, Laboratory of Viral Diseases, NIH, USA. To generate stable cell lines, Vero cells were transfected with CD32a plasmid, followed by selection in the presence of 50 mg/mL G418. This is an unpublished information. We have added this information in the revised manuscript (Line no. 148-149, page no. 7).

(2) Lines 256-257 and Fig. 2A: Authors should repeat the ADE assay by reducing viral tier in the control.

**Response:** We have repeated the ADE assay using HB112 mAb by reducing the input DENV-2 virus at MOI=0.05 in Vero and Vero CD32a cell lines. We observed peak-enhancement of DENV-2 infection at 0.1µg/ml HB112 concentration as reported by others (Moi et al, 2011 J Infect Dis. <https://academic.oup.com/jid/article/203/10/1405/817308>). Please find the image below for your reference.

(3) The ADE pattern of dose response curves in Figs. 3A and 3B did not match the micrographs in Figs. 2A and 2B, although there were some differences in the concentration of HB112. Since the gap will make it difficult for readers to understand the ADE assay, these data should be correlated.

**Response:** As pointed out correctly, the dose response curves in Fig 3A and 3B look different than the representative image indicated in Fig 2A and 2B due to the differences in the range of concentration of the antibody used in experiments. We have added the new figure panel, 2C of the same concentration as shown in Figures 3A and 3B.

A preliminary experiment of the use of different cell seeding densities showed comparable levels of DENV-2 infection enhancement in U937 and K562 cells (Figure 2B). However, when multiple experiments were performed using the two cell lines, U937 and K562 at 20,000 cells/well seeding density, K562 appears to be superior to U937 cells. This data is already presented in figures 3A and 3B. We have replaced figures 2A and 2B in the revised manuscript.

(4) Figs. 4E and 4F suggested that K562 cells likely showed higher fold enhancement than U937 cells. However, micrographs in Figs. 2A and 2B indicated that U937 cells clearly displayed stronger ADE (fold enhancement) than K562 cells, which suggests that U937 cells may be more sensitive to ADE assay compared with K562 cells. This phenomenon is opposite to Figs. 4E and 4F. What is the cause of this difference? Did the difference between serum and MAb make this? Authors need to consider these points.

**Response:** We have presented a representative image in Figs. 2A and 2B that is giving contrasting results, with U937 being more sensitive to ADE assay than K562 cells. Sorry about that.

We have revised figures 2A, and 2B for better clarity. Data shown in both Figs.3 and 4 indicates that HB112 (monoclonal antibody, Fig.3) or donor samples (Fig.4) showed a higher degree of fold-enhancement towards DENV-2 and DENV-4 infections when K562 cells were used.

Minor comments

(1) How many cells (cells/well) did you use in the assays of Fig. 2A? (20,000 cells/well)

“CELLS/ML” should be “cells/well” in Fig. 2B

**Response:** We have used 20,000 cells/well in fig.2A. We have added this information in figure legend. Thank you for pointing out the typo-error in Fig.2B. We have revised figure 2B as suggested.

**Response to the Reviewer 2:**

Reviewer #2: 1. The manuscript titled "Comparative evaluation of methods for the measurement of antibody-dependent enhancement of dengue virus infection in FcγRIIa expressing cell lines" demonstrate the utilization of different cell lines for ADE analysis. The title itself is little misleading. Actually, the authors have performed similar methodology using different cell lines, so it may be corrected.

**Response:** We've made changes to the title as suggested.

2. In the manuscript, it is concluded that the infection-ADE assay using K562 cells is the most suitable method to determine the enhancement of infection at sub-neutralizing dilutions, however it is directly corelated with the amount of FcγRIIa expression in the cells, which is quite obvious.

**Response:** Several FcγRIIa expressing cell lines such as K562, U937, THP-1, BHK-21, and CV-1 cells have been used to measure the ADE response. Cell lines such as K562, U937, and THP-1 cells naturally express FcγRIIa receptor alone or in the presence of other receptors such as FcγRI. While other cell lines such as BHK-21 or CV-1 cells have been genetically modified to express the FcγRIIa receptors. These modified cell lines resulted in lesser expression of FcγRIIa as depicted in Figure 1A for Vero-CD32a cells. Simultaneous experiments in two cell lines, U937 and K562 provide direct evidence of the positive correlation of FcγRIIa expression with enhancement of infection in ADE assay.

We have discussed this point in the revised manuscript (Line no. 567-573, page no. 26).

3. The cross-reactivity should be analyzed with all the four DENV serotypes or at least using VLPs.

**Response:** We have performed neutralization and ADE assay against all four serotypes and presented the data in Table 3 in the revised manuscript to address the cross-reactivity phenomena in dengue infections.

4. Discussion part of the manuscript is written well, however, Abstract, Introduction, methodology, and result sections need revision in terms of clarity.

**Response:** We have tried our best to improve the text in the abstract, introduction, methodology, and results sections in the revised manuscript.
