# Supplementary material for: Evaluation of methods for the measurement of antibody-dependent enhancement of dengue virus infection using different FcγRIIa expressing cell lines
Source: PLoS One. 2025 Aug 29;20(8):e0331320. doi: 10.1371/journal.pone.0331320 (PMC12396659; doi:10.1371/journal.pone.0331320)
Supplement: Attachment — Submitted filename: Response to reviewers_02Jul2025.docx [file pone.0331320.s002.docx]

Dear Editor and Reviewers,

Thank you for your valuable feedback on our manuscript titled "Comparative evaluation of methods for the measurement of antibody-dependent enhancement of dengue virus infection in FcγRIIa expressing cell lines" (Manuscript ID: PONE-D-24-39295 PLOS ONE). We appreciate the time and effort you have invested in reviewing our work. We have carefully considered all the comments and have made significant revisions to address them.

**Response to the Academic editor:**

1. It remains unclear whether Vero-CD32a cells exhibit ADE for dengue virus. Please clarify this point and provide an explanation for the results shown in Figures 7 and 8.

**Response:**

In the current manuscript, we attempted to evaluate different cell lines and different test formats for their ability to exhibit ADE using the same set of samples. In the ADE-infection assay, initially only two cell lines, K562 and U937, were compared. We have performed ADE-infection assay using Vero-CD32a, and the data were compared against the other two cell lines (revised Figure 4). Our data demonstrate that the enhancing activity was seen with K562 and U937 cells, whereas Vero-CD32a cells were unable to exhibit ADE (revised Figure 4).

When FRNT was performed simultaneously in Vero and Vero-CD32a cells, individual samples did show enhancing activity in Vero-CD32a cells, but the magnitude of fold enhancement was minimal, up to 2-fold in Vero-CD32a cells (Figures 7 and 8). When we did paired analyses of GMT titres employing Wilcoxon signed rank test in blood donor samples, no significant differences were observed (revised Figure 7). Similarly, comparable DENV-2 titres and significantly lower DENV-4 titres were obtained in Vero-CD32a than in Vero cells when secondary dengue samples were analysed (revised Figure 8).

Thus, the infection-ADE assay is a suitable method for ADE assessment. Comparison of FRNT50 titres in Vero and Vero-CD32a cells did not show distinct enhancing activity, probably due to the samples having high neutralizing activity. We have revised the manuscript to bring clarity in the manuscript.

**Response to the Reviewer 1:**

Reviewer #1: Authors have responded to most of the reviewer’s comments. However, still I do not understand one point. They need to indicate clearly whether Vero-CD32a cells display ADE or not. Their present statement will make readers confused. Several sentences in the manuscript are contradictory (see below). Especially, Figs. 8C and 8D simply showed result of FRNT, thus the authors’ statement regarding infection-enhancing activity was misleading (Lines 465-466).

Lines 283-284: “no distinct enhancement pattern was observed at any HB112 concentrations in Vero-CD32a cells to No Ab control wells”.

Lines 465-466: “This data suggests that low infection-enhancing activity was noted in Vero-CD32a cells.”

Lines 627-629: “It was observed that follow-up samples collected after 10 years post-vaccination showed enhancement of infection in Vero-CD32a cells when compared to sera collected at day 208 post-vaccination”.

**Response:** We have revised the contradictory statements. Individual samples did show enhancing activity in FRNT method, however, when a non-parametric Wilcoxon signed rank test was performed, titres were found comparable in both Vero and Vero-CD32a cells against DENV-2 (Fig. 8C), but not with DENV-4 viruses (Fig. 8D). Enhancing activity is influenced by DENV serotypes and we have discussed this point in the revised manuscript (line no. 628-637, page no. 28). We have revised the line no. 283-284, 465-466 and line no. 627-629 for better clarity.

**Response to the Reviewer 3:**

1. The manuscript reports that Vero-CD32a cells showed no distinct ADE activity, even at lower MOIs (Figure 2A), yet later states that some secondary dengue samples exhibited infection-enhancing activity in Vero-CD32a cells (Table 2). This contradiction is not addressed, leading to confusion about the utility of Vero-CD32a cells for ADE measurement.

**Response:** As indicated in Figure 2A, Vero-CD32a did not demonstrate any enhancing activity at any concentrations of HB112 (4G2) antibody, even at lower MOI of 0.05. Moi et al (2013) demonstrated that monoclonal antibody concentration but not the challenge virus titres influence the ADE activity of either monoclonal antibodies or dengue patients’ serum samples.

In fact, we followed Moi et al (2013) methodology to measure the *in-vitro* ADE activity of human serum samples by conventional plaque assay using FcγR-expressing cells. We used FRNT data to re-analyze in terms of virus titres at different dilutions of samples and compared the titres in Vero and Vero-CD32a cells, which led to contradictory results as shown in Table 2.

We have removed Table 2 from the revised manuscript. For ADE measurement, only two methods were evaluated and compared (1) ADE-infection assay from the culture supernatants, and (2) simultaneous performance of FRNT in Vero and Vero-CD32a cells throughout the manuscript, and results were discussed.

Secondary dengue samples showing infection-enhancement activities to a lower extent using Vero-CD32a cells were discussed in the revised manuscript (line no. 628-637, page no. 28).

2. The manuscript concludes that K562 cells are superior for ADE assays due to higher FcγRIIa expression and lack of viral replication in the absence of antibodies. However, it does not explore the mechanistic reasons for this superiority, such as differences in FcγRIIa signaling, internalization efficiency, or cell-specific responses to DENV. The claim that K562 cells only express FcγRII is not supported by a reference or experimental validation.

**Response:** K562 cells predominantly express FcγRIIa receptor with an activating cytoplasmic ITAM motif. Boonnak et al (2013) demonstrated that ADE of dengue virus infection was significantly abrogated in K562 cells when either monoclonal antibodies blocking the FcγRIIa surface expression or siRNA treatment to knockdown FcγRIIa expression were used. We have provided this information in the revised manuscript (line no. 553-562, page no. 25).

3. The manuscript reports significantly lower neutralizing antibody titers in Vero-CD32a compared to Vero cells for DENV-2 (Line 430) but not for DENV-4 (Line 435). This contradicts prior studies that found lower neutralizing titers in FcγRIIa-expressing cells for all serotypes due to ADE. The authors do not explain why DENV-4 behaves differently or reconcile their findings with existing literature.

**Response:** We have explained the contradictory results obtained in our study with prior research articles (Line no. 616-637, Page no. 28).

4. The optimization of the ADE assay in Vero-CD32a cells is inadequate. The manuscript notes that "No Ab control wells were fully saturated with infection at 1 MOI" (Line 280), and even at a lower MOI (0.05), no ADE was observed (Line 283). This suggests that the assay conditions were not optimized for Vero-CD32a, potentially skewing the comparison with K562 and U937 cells. Additionally, the cut-off for fold-enhancement (1.8 for Vero-CD32a, 10 for K562/U937) differs between assays without justification.

**Response:** Since, DENV infection enhancement activity could be influenced by input virus MOI, antibody concentration, and virus serotype, we optimized the ADE assay using DENV-2 infection at different MOI and HB112 (4G2) antibody concentrations in K562, U937, and Vero-CD32a cell lines. Distinct enhancement of DENV-2 infection was seen in K562 and U937 cells but not in Vero-CD32a cells (Revised Figure 4).

As described earlier, for the evaluation of virus titres at different dilutions using FRNT data, cut-off value of 1.8 for fold-enhancement was used following Moi et al (2013) paper. We have removed this analysis from the revised manuscript for better clarity.

5. The manuscript briefly mentions the limitation of not knowing the infecting serotypes for secondary dengue patients (Lines 653-656) but does not discuss the implications of this gap. For example, cross-reactive antibodies could skew ADE results, particularly in hyperendemic areas. Additionally, the study does not address the potential impact of FcγRIIa polymorphisms (e.g., R131H), which are known to influence ADE.

**Response:** We have discussed these points in the revised manuscript (line no. 676-696, line no. 30).

6. The presentation of results, particularly in Figures 7 and 8, is confusing. For example, Figure 7A shows a significant difference in FRNT50 titers for DENV-2 between Vero and Vero-CD32a cells, but the text does not explain why only 33% of samples showed a reduction (Line 426). Figure 8C-D reports comparable titers for DENV-2 but a significant difference for DENV-4, without clear justification. Additionally, the use of geometric mean titers obscures individual sample variability.

**Response:** We have revised Figures 7 and 8, a non-parametric Wilcoxon signed rank test was performed to obtain statistical value, donor samples did not exhibit enhancing activity in Vero-CD32a cells (revised Figure 7).

There is a significant difference in titres against DENV-4 in secondary dengue samples, but not with DENV-2 serotype. There are multiple factors involved like sample type, previous primary or secondary DENV immunity, quantity of cross-reactive neutralizing antibodies, and serotype-specific differences in neutralizing antibody response that could lead to differences in serotype-specific titres. We have provided justification in the revised manuscript (line no. 618-637, page no. 28).
